# Mutation in *XPO5* causes adult-onset autosomal dominant familial focal segmental glomerulosclerosis

**DOI:** 10.1186/s40246-022-00430-y

**Published:** 2022-11-12

**Authors:** Hafiz Muhammad Jafar Hussain, Yikai Cai, Qinjie Weng, Jun Tong, Ayesha Aftab, Yuanmeng Jin, Jian Liu, Shuwen Yu, Zhengying Fang, Wen Du, Xiaoxia Pan, Hong Ren, Jingyuan Xie

**Affiliations:** 1 Department of Nephrology, Institute of Nephrology, Shanghai Ruijin Hospital, Shanghai Jiao Tong University, School of Medicine, 197 Ruijin Er Road, Shanghai, 200025 China; 2 Department of Molecular and Human Genetics, Baylor College of Medicine, Houston, TX 77030 USA; 3 Department of Biological Sciences, International Islamic University, Islamabad, 44000 Pakistan

**Keywords:** FSGS, Whole-exome sequencing, *XPO5* gene, Adult-onset, Autosomal, Dominant

## Abstract

**Background:**

Focal and segmental glomerulosclerosis (FSGS) is a histological pathology that characterizes a wide spectrum of diseases. Many genes associated with FSGS have been studied previously, but there are still some FSGS families reported in the literature without the identification of known gene mutations. The aim of this study was to investigate the new genetic cause of adult-onset FSGS.

**Methods:**

This study included 40 FSGS families, 77 sporadic FSGS cases, 157 non-FSGS chronic kidney disease (CKD) families and 195 healthy controls for analyses. Whole-exome sequencing (WES) and Sanger sequencing were performed on probands and family members of all recruited families and sporadic FSGS cases.

**Results:**

Using WES, we have identified a novel heterozygous missense variant (c.T1655C:p.V552A) in exportin 5 gene (*XPO5*) in two families (FS-133 and CKD-05) affected with FSGS and CKD. Sanger sequencing has confirmed the co-segregation of this identified variant in an autosomal dominant pattern within two families, while this variant was absent in healthy controls. Furthermore, the identified mutation was absent in 195 ethnically matched healthy controls by Sanger sequencing. Subsequently, *in silico* analysis demonstrated that the identified variant was highly conservative in evolution and likely to be pathogenic.

**Conclusions:**

Our study reports an adult-onset autosomal dominant inheritance of the *XPO5* variant in familial FSGS for the first time. Our study expanded the understanding of the genotypic, phenotypic and ethnical spectrum of mutation in this gene.

**Supplementary Information:**

The online version contains supplementary material available at 10.1186/s40246-022-00430-y.

## Introduction

Focal segmental glomerulosclerosis (FSGS) is characterized by focal segmental sclerosis with foot process effacement histologically. The causes of FSGS can be intrinsic or extrinsic. Based on mechanism, FSGS is classified into four types including primary FSGS, genetic FSGS, secondary FSGS and FSGS of undetermined cause. Secondary FSGS can be the result of systemic conditions, i.e., hypertension or obesity, viral infections, the conditions causing a reduction in the number of functioning nephrons like surgical ablation or unilateral renal agenesis, and in other kinds of primary glomerulonephritis [1, 2]. The pathogenesis of FSGS is not well studied yet. However, the numbers of FSGS-associated genes identified using high-throughput sequencing techniques have been increasing in recent years [3].

The important cellular processes occur in nucleoplasm and cytoplasm which need bidirectional trafficking of molecules between these compartments, but a double-membrane nuclear envelope restricts the free movements of molecules [4]. The nuclear envelope contains many nuclear pore complexes (NPCs) which are embedded in the nuclear membrane. Nucleoporins (Nup) are the small units of NPCs, which are well organized and are the pathway for facilitating the trafficking between the nucleoplasm and cytoplasm [5]. Moreover, small molecules can pass through these channels by passive diffusion, but importins and exportins are required for the transportation of macromolecules that are larger than ~ 40 kD [6].

The importins and exportins, are not only responsible for nucleocytoplasmic transport but are also involved directly or indirectly in various cellular processes, such as cell differentiation [6]. During development, a differential expression of exportins (XPO1, XPO5, XPO6 and XPO7) was noticed among different cell types and tissues (https://www.genecards.org/). Moreover, variants in exportins have been associated with several diseases such as cancer, autism, schizophrenia, and steroid-resistant nephrotic syndrome (SRNS) [7–10].

Exportin 5 (*XPO5*) plays a specific role in the nucleocytoplasmic transport of double-stranded RNA binding protein Staufen2 and pre-miRNAs, which are important in the regulatory cascade of kidney development, maintenance of renal function, and progression of renal diseases [11–13]. Besides, *XPO5* interacts with several nucleoporins such as NUP107, NUP93 and NUP205 directly or indirectly during the cargo process, which has been associated with kidney diseases [14]. Braun DA et al. demonstrated that XPO5 is present in developing podocytes that are positive for the podocyte nuclear marker WT1 and in adult rat glomeruli, it is localized in podocytes with synaptopodin [14]. They also demonstrated that loss of function mutations in NUP93 identified in patients affected with SRNS either disrupt NPC integrity or repudiate the interaction of NUP93 with importin7 or SMAD4 in HEK293 cells. Moreover, there was a defect in SMAD signaling in all individuals, with mutations in NUP93, suggesting SMAD signaling as the relevant pathogenic pathway. They confirmed that the individuals with a defective SMAD signaling pathway, caused by NUP93 variants, were affected with SRNS in their early ages. Taking all together, disruption in XPO5 can cause defective SMAD signaling and hence cause kidney dysfunction.

More than 50 genes have been found to be associated with familial FSGS in autosomal dominant (AD), autosomal recessive (AR) or X-linked inheritance patterns [3, 15]. However, in Asian population a very low rate of exportin genes variants was reported, suggesting that new genes or variants may cause FSGS pathogenesis [3]. In this study, we included 40 FSGS families, 77 sporadic FSGS cases and 157 non-FSGS chronic kidney disease (CKD) families for analyses. We identified a novel heterozygous missense variant of *XPO5* in two of the FSGS families. Sanger sequencing confirmed the co-segregation of *XPO5* variant in an autosomal dominant pattern within the two families while this variant was absent in 195 ethnically matched healthy controls. In silico analysis demonstrated that this identified variant was highly conservative and likely to be pathogenic. Our study reported an adult-onset autosomal dominant inheritance of *XPO5* variant in familial FSGS for the first time. The newly identified variant expands the mutational and phenotypic spectrum as well as the ethnic occurrence of disorders previously associated with this gene. Our findings will provide an insight for nephrologists to consider such rare genetic causes in complex phenotypes regardless of ethnicity.

## Results

### Clinical and pathological characteristics of patients with *XPO5* variant

**Fig. 1:**
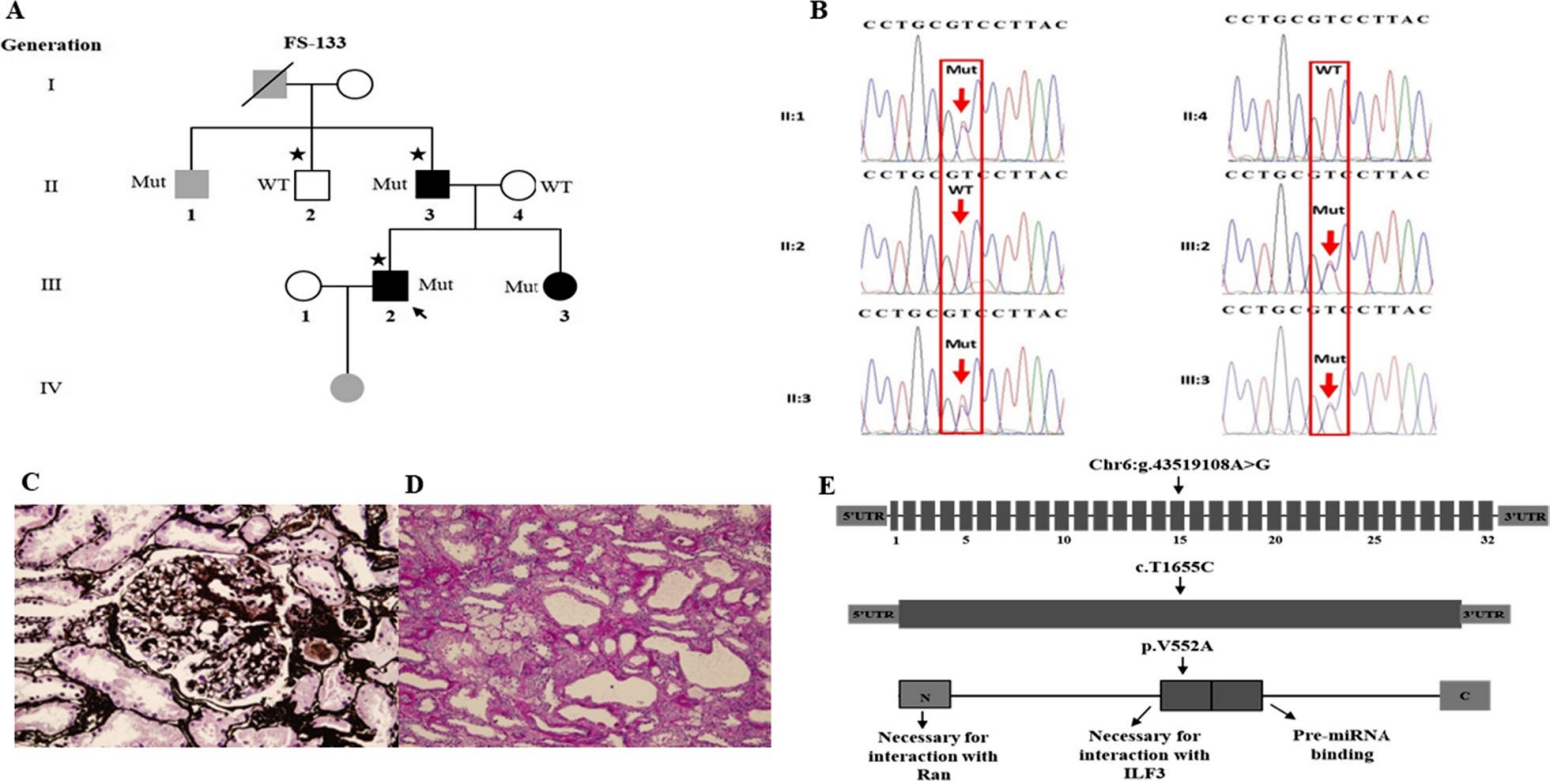
Identification of a heterozygous *XPO5* missense variant in familial FSGS (FS-133). **A** Pedigree of the family (FS-133). Symbols are represented as: males (squares), females (circles), affected individuals having kidney related problem (black), phenotype unknown (gray), unaffected individuals (blank), star (individuals selected for whole-exome sequencing), and arrowhead (proband). **B** Sanger sequencing of mutant *XPO5* (c.T1655C, p.V552A) of II: 1, II:3, III:2 and III:3 and wild type for II:2 and II:4. **C** Jones stain (400X) showed one nearly normal glomerulus. **D** Periodic acid–Schiff (PAS) stain (400X) showed foam cells (white arrow) in renal interstitium. **E** The *XPO5* variant chr6: g.43519108A > G is supposed to cause a T > C substitution at complementary DNA nucleotide 1655 and introduce a substitution codon at codon 552, resulting in a missense variant p.V552A in a predicted protein of 1204 amino acids. Information of protein domains is from UniProt (Q9HAV4). Mut, mutant; WT, wild type

**Fig. 2:**
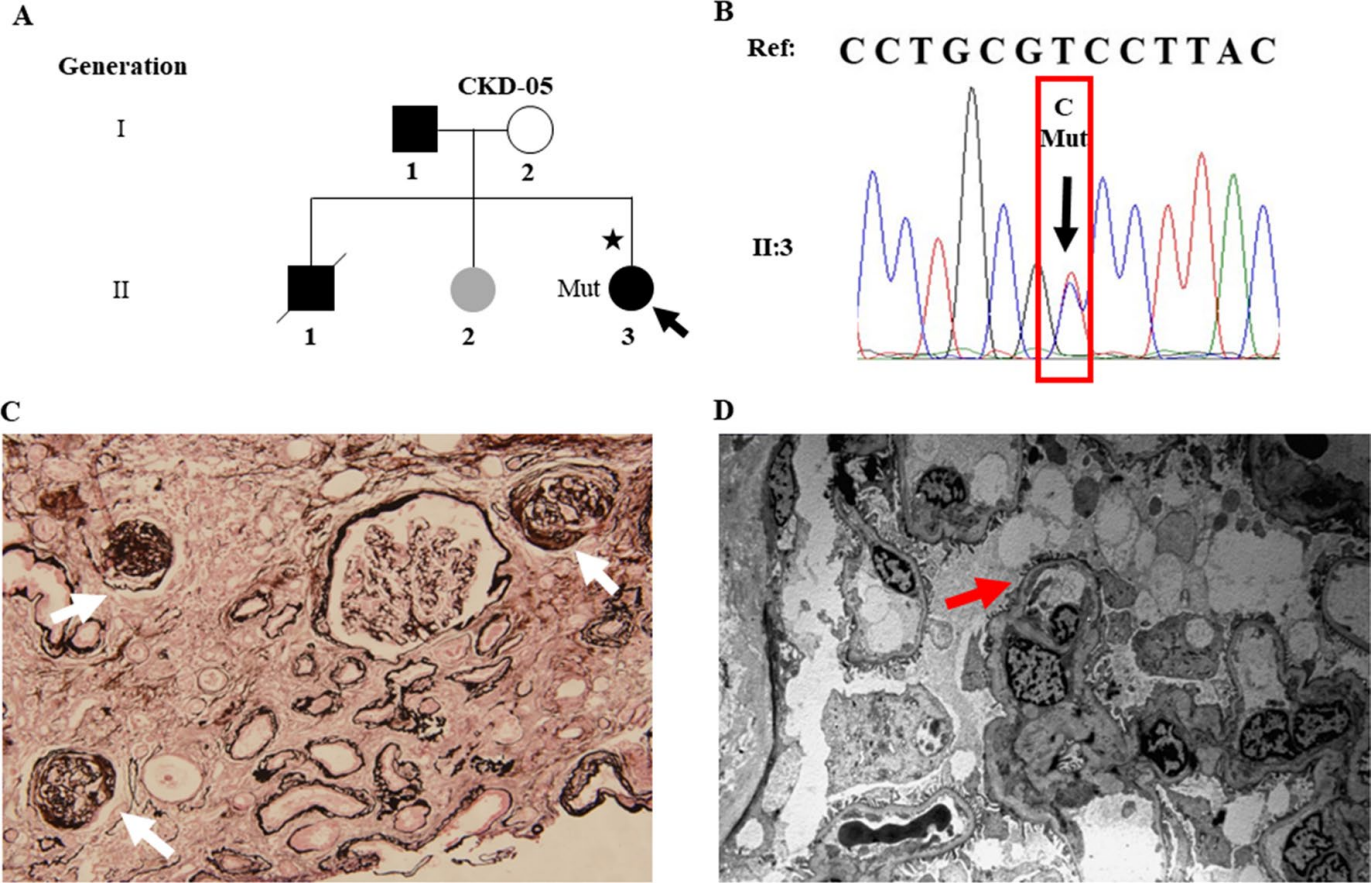
Identification of a heterozygous *XPO5* missense variant in familial FGGS (CKD-05). **A** Pedigree of the family (CKD-05). Symbols are represented as: males (squares), females (circles), affected individuals having kidney related problem (black), phenotype unknown (gray), unaffected individuals (blank), star (individuals selected for whole-exome sequencing), and arrowhead (proband). **B** Sanger sequencing of mutant *XPO5* (c.T1655C, p.V552A) of II: 3. **C** Jones stain (200X) showed three glomeruli with global sclerosis (white arrows). **D** Electron micrographs (2500X) showed partial fusion of foot processes (red arrow). Mut, mutant; WT, wild type

**Fig. 3:**
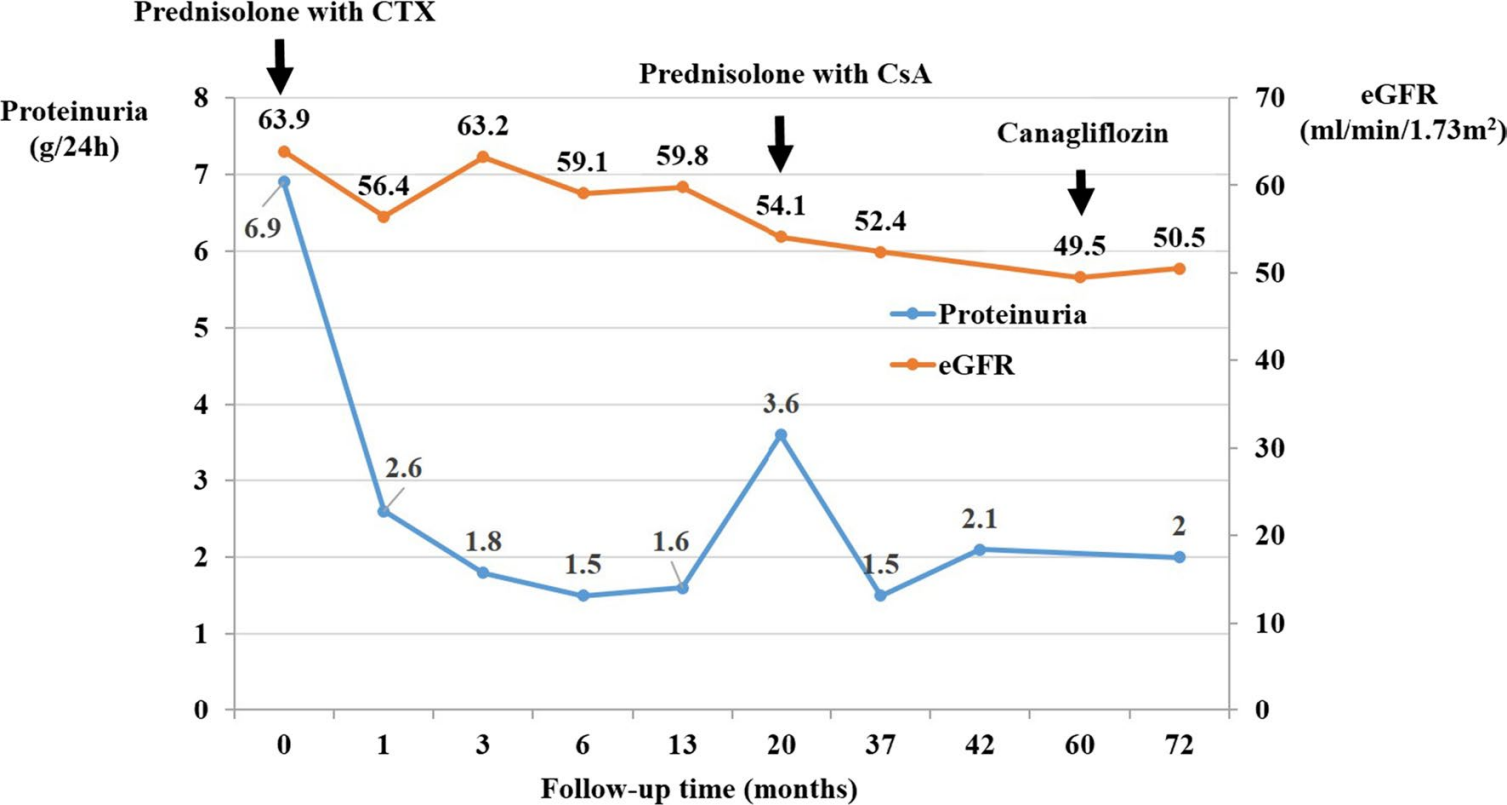
Treatment and follow-up of proband (III:2) of family (FS-133). Proteinuria and eGFR of proband (III:2) of family FS-133 at baseline during treatment and follow-up. CTX, cyclophosphamide; CsA, cyclosporine A

In the selected families related to *XPO5* variant, the average age of patients was 49.2 years and the average disease onset age was 29.5 years. The pathogenic variant of *XPO5* was detected in two families (FS 133 & CKD-05) with the familial CKD (Fig. 1A & 2A). Primarily, the proband (III:2) of family FS-133 (Fig. 1A) underwent a kidney biopsy because of nephrotic range proteinuria (4 g/24 h) and renal insufficiency (serum creatinine 107 μmol/L) when he was 23 years old. He was diagnosed with FSGS by renal biopsy, and unfortunately biopsy images were not available when he was initially diagnosed. Renal biopsy showed 1–2 glomeruli with focal sclerosis among 8 glomeruli. Mild interstitial fibrosis and renal tubular atrophy were noted. Immunofluorescence microscopy revealed non-specific patchy depositions of immunoglobulin M (IgM) and complement C3 in one glomerulus. He was treated with prednisolone and immunosuppressive agent (Tripterygium wilfordii). When he was 42 years old, he was admitted to our hospital again. He presented with a nephrotic range of proteinuria (4–6.9 g/24 h) without nephrotic syndrome (serum albumin 37 g/L). He was found to have increased urinary albumin/creatinine ratio (457.33 mg/mmol), elevated serum creatinine levels (126 μmol/L) (EPI-eGFR 60.2 ml/min/1.73m^2^), increased uric acid level (401 μmol/L) and hypertension (147/90 mmHg) (Table 1). Repeated renal biopsy was performed (Fig. 1C, 1D), and two of four glomeruli were global sclerosis with mild mesangial cell proliferation under light microscopy. Severe interstitial fibrosis, renal tubular atrophy and scattered inflammatory cell infiltration were noted. Immunoglobulins and complements including IgG, IgA, IgM, C3, C4, C1q, light chain κ and λ staining were all negative. Then, prednisolone in combination with intravenous cyclophosphamide (CTX) was initiated. A total dose of CTX was up to 8.6 g, which resulted in decreased proteinuria to 1.5 g/24 h at first but increased to 3.6 g/24 h at the 20th month of follow-up. Thus, 150 mg/24 h of Cyclosporine A (CsA) was started and proteinuria gradually decreased to about 2 g/24 h, then prednisolone and CsA were gradually reduced for 2 years. Lastly, 18 months ago from now, canagliflozin was initiated and his proteinuria was maintained at about 2 g/24 h till now. Overall, his proteinuria decreased by more than 50%. During follow-up, EPI-eGFR was slightly decreased to about 50 ml/min/1.73m^2^ (Fig. 3). Besides, the proband’s father (II:3) was affected with proteinuria [urinary albumin/creatinine ratio (ACR) 74.03 mg/mmol], microscopic hematuria with elevated serum creatinine level (138 μmol/L) (EPI-eGFR 46.5 ml/min/1.73m^2^) and increased uric acid level (611 μmol/L) at the age of 63. Moreover, the proband’s uncle (II:1) presented with microscopic hematuria, but the results showed normal creatinine and no proteinuria as tested by urine routine test at the age of 60. Therefore, his phenotype was defined as unknown. The proband’s sister (III:3) showed proteinuria (urinary ACR 12.61 mg/mmol) at age of 37 with normal renal function (eGFR 97 ml/min/1.73m^2^). All the clinical characteristics of family FS-133 are given in Table 1.

**Table 1:** Clinical characteristics of families having *XPO5* variant

Family ID	Individual ID	Affected	Sex	Age (Years)	Age of onset (years)	KB	Hematuria	UP (g/24 h or qualitative)	Scr (μmol/L)	eGFR (ml/min/1.73m^2^)	BP (mmHg)	UA (μmol/L)	ACR (mg/mmol)
FS-133	III:2	2	M	42	23	FSGS	Negative	6.9	126	60.2	147/90	401	457.33
FS-133	II:1	0	M	60	–	–	2 +	–	55	107.7	–	297	< 2.5
FS-133	II:2	1	M	51	–	–	Negative	–	79	98.8	–	–	–
FS-133	II:3	2	M	63	56	–	1 +	3 +	138	46.5	–	611	74.03
FS-133	II:4	1	F	64	–	–	Negative	–	74	73.8	–	265	3.01
FS-133	III:3	2	F	37	37	–	Negative	–	69	97	–	225	12.61
CKD-05	II:3	2	F	24	24	FGGS	2 +	0.179	332	15.9	110/70	512	–
CKD-05	I:1	2	M	60	60	–	Negative	Negative	186	33.1	130/80	446	–
CKD-05	I:2	1	F	59	–	–	Negative	Negative	56	98	–	351	–
CKD-05	II:2	0	F	32	–	–	3 ~ 7/HP	Negative	68	102.3	–	377	–
CKD-05	III:2	2	M	Died of ESRD at 20	20	–	–	–	–	–	–	–	–

Affected, unaffected = 1, affected = 2, phenotype unknown = 0, *KB* Kidney biopsy, *FSGS* Focal segmental glomerulosclerosis, *FGGS* Focal global glomerulosclerosis, *UP* Urine protein, *Scr* Serum creatinine, *BP* Blood pressure, *UA* Uric acid, *HP* High power field, “-”, not available

The proband (II:3) from another CKD family (CKD-05) presented with mild proteinuria (0.179 g/24 h), significantly increased serum creatinine level (332 μmol/L, eGFR 15.9 ml/min/1.73m^2^) and elevated uric acid level (512 μmol/L) at the age of 24 years (Table 1). She was diagnosed with focal glomerulosclerosis by renal biopsy (Fig. 2C, 2D). Under the light microscopy, the specimen contained 14 glomeruli out of which 5 glomeruli showed global sclerosis. No obvious mesangial proliferation and thickening of GBM were observed, while mild interstitial fibrosis, renal tubular atrophy and scattered inflammatory cells infiltration were noted. Moreover, electron microscopy (EM) of the patient showed partial fusion of foot processes. Besides, immunoglobulins and complements including IgG, IgA, IgM, C3, C4 and C1q, were all negative. She was eventually diagnosed with focal global glomerulosclerosis (FGGS). However, FSGS could not be ruled out because of limited number of glomeruli available. Additionally, her father (I:1) presented with increased serum creatinine level (186 μmol/L, EPI-eGFR 33.1 ml/min/1.73m^2^), elevated uric acid level (446 μmol/L) and hypertension (130/80 mmHg). Moreover, her sister (II:2) exhibited microscopic hematuria (3–7/HP) with urinary dysmorphic red blood cells of 82% and a minor increase in uric acid level (377 μmol/L) at the age of 32. She had normal serum creatinine and no proteinuria, so her phenotype was defined as unknown. Moreover, the proband’s brother (II:1) died of ESRD at the age of 20 years (Fig. 2A and Table 1).

### Genetic analysis of patients with *XPO5* variant

We identified a causative heterozygous variant in exon 15 (NM_020750: exon15:c.T1655C:p.V552A) of *XPO5* using WES*,* accounting 2.5% (1/40) in FSGS families (FS-133), and 0.64% (1/157) in CKD families (CKD-05, Fig. 1A and Fig. 2A). However, it was found absent among sporadic FSGS patients. Furthermore, the Sanger sequencing validated the segregation of the identified variant with diseased phenotype in both families. In the FS-133 family, the heterozygous mutation was detected in four individuals with three affected (II:3, III:2, III 3) and one unknown phenotype (II:1), while the control (II:2) from this family was wild type (Fig. 1B). The proband (II:3) of CKD-05 family was also heterozygous for this identified variant (Fig. 2B). Furthermore, the identified variant was absent in 195 ethnically matched healthy controls as shown by Sanger sequencing. The MAF of the identified variant in gnomAD was 0.0006058. Using different databases, including Human Gene Mutation Database, ClinVar and a literature survey via PubMed, the gene mutation was searched and it was noted that this variant has not been previously reported to the best of our knowledge (Fig. 3).

### In silico structural prediction of *XPO5* variant

**Fig. 4:**
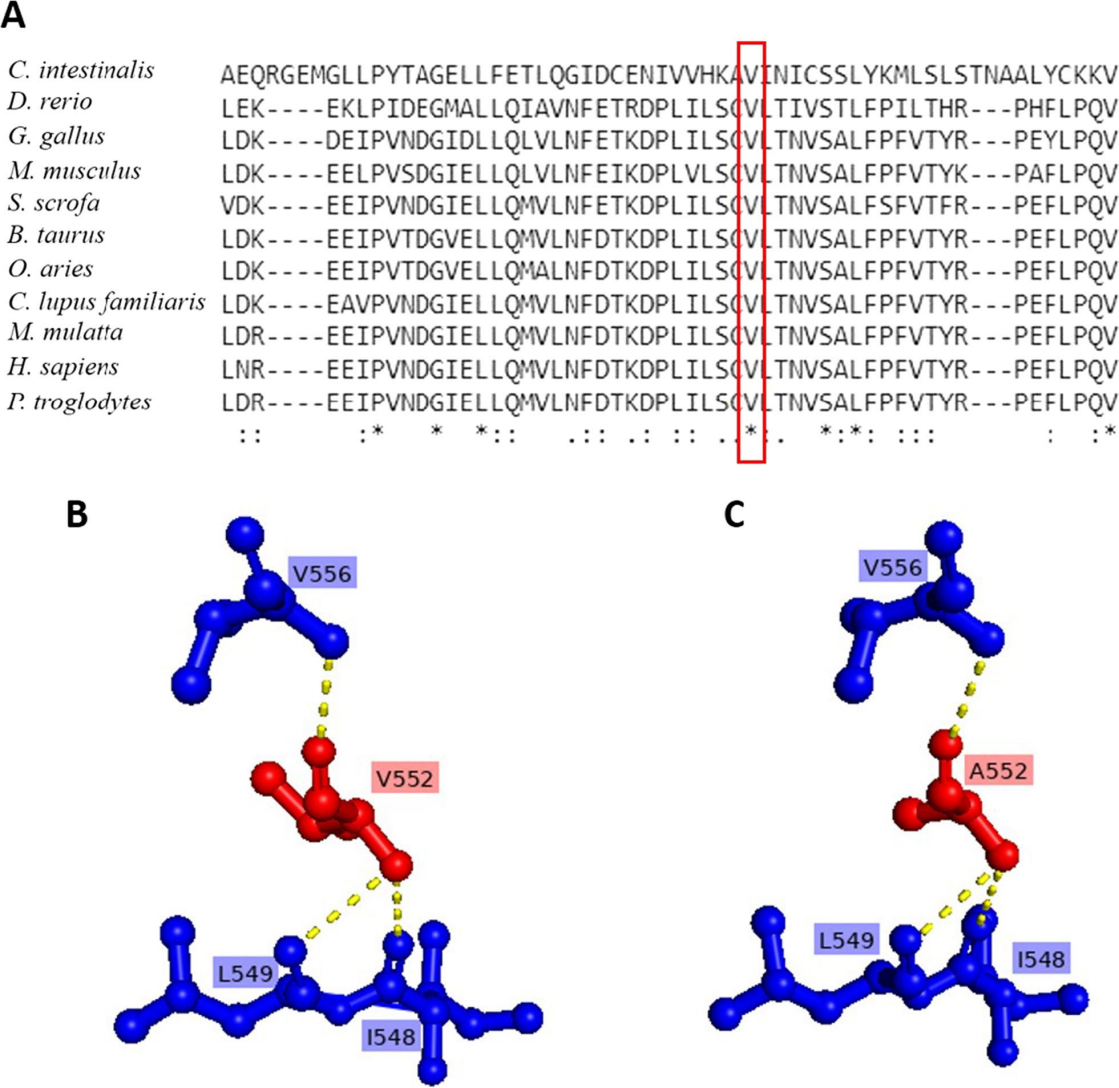
*In silico* analysis of the identified *XPO5* missense variant. **A** Evolutionary conservation analysis of identified XPO5 variant; **B** the predicted structure of wild-type (Valine) residue of XPO5; **C** the predicted structure of mutant residue (p.V552A). The mutant XPO5 (V552A) effect on hydrogen bonding in protein structure. Red residue are sites of variant and wild type, while interacting hydrogen-bonded residues are shown in blue. The yellow dotted lines are displaying hydrogen bonding

The in silico analysis tools MutationTaster, Polyphen-2 and FATHMM-XF predicted p.V552A as deleterious, probably damaging and pathogenic. The combined annotation-dependent depletion (CADD) showed a PHRED score of 24.3, predicting that the identified variant can be pathogenic by affecting the structural properties of the protein. CADD-SV scores on the PHRED scale range from 0 (potentially benign) to 48 (potentially pathogenic). Moreover, pLI score of *XPO5* is 1, which is an indication of the intolerance for the missense variant. The pLI score reflects the tolerance of a given gene to the loss of function on the basis of the number of protein-truncating variants including the frameshift, splice donor and acceptor, and stop-gain variants referenced for this gene in control databases weighted by the size of the gene and the sequencing coverage[16]. Additionally, ACMG criterion has also classified this variant as “likely pathogenic” (Additional file 1: Table S1). The evolutionary conservation of mutation site was analyzed by the multiple sequencing alignment which shows the wild-type amino acid is highly conserved (Fig. 4A); therefore, any mutation at this site may lead to a damaging impact on XPO5 expression.

“*” indicates a single fully conserved residue; “:” indicates conservation between similar groups, “.” indicates weak conservation between different groups.

Additionally, the HOPE analysis showed that the size of substituted residue (alanine) is smaller than the wild-type (valine) residue, hence leaving a space in the core of the protein. Moreover, the variant is located within a stretch of residues annotated in UniProt as a special region that is necessary for interaction with ILF3. The differences in wild-type and mutant amino acid properties can disturb this region and its function(s). Besides this, Missense3D analysis has predicted that this variant resulted in the expansion of cavity volume by 53.784 Å^3 and is buried in the core of protein that might disturb the core structure of the domain (Fig. 4B and 4C). These in silico findings indicate that p.V552A is a deleterious variant, which might have a damaging impact on protein structure and function.

## Discussion

FSGS is a leading cause of end-stage kidney disease. Genetic factors significantly contribute to the pathogenesis of the diseases. To date, mutations in more than 50 genes have been reported to be involved in FSGS [17]. This number is rising rapidly due to the advancement of sequencing technologies during the last two decades. It is found that mutations involving the slit membrane components *(NPHS1, NPHS2, COL4A3/A4/A5, *etc*.*), collagen genes (*COL4A3/A4/A5, *etc*.*), podocyte cytoskeleton *(ACTN4, INF2*, etc.), calcium channel protein (*TRPC6*, etc.), mitochondrial function (*ADCK4, COQ2, COQ6*, etc.) and transcription factors (*WT1, PAX2*, etc.) [18].

The overall prevalence of FSGS varies in different populations, for example in African Americans it is the most common cause of glomerulonephritis. Its prevalence also varies across different regions of the same country; in China, it varies from 3 to 6 and 14 to 16% of all kidney biopsies in North and South China, respectively [19]. The differences in its prevalence and frequency of familial cases of FSGS proclaim its strong genetic association [20]. In this study, we identified a novel heterozygous missense variant (Chr6: g.43519108A > G, c.T1655C, p.V552A) in *XPO5* using WES and Sanger sequencing*.* This variant was found to be pathogenic in two unrelated Chinese families of adult-onset familial FSGS and FGGS. *XPO5* is present on the reverse strand of chromosome 6 and our identified variant (Chr6: g.43519108A > G) corresponds to c.T1655C at complementary DNA level and p.V552A on protein level, respectively.

In our study, we identified a novel heterozygous variant (c.T1655C:p.V552A) in XPO5. The average onset age of our patients was 29.5 years. The proband of family FS-133 was presented with nephrotic range proteinuria (4 g/24 h) and renal insufficiency. Renal biopsy showed FSGS. During follow-up, his proteinuria decreased by more than 50%. His renal function was slightly decreased. The other proband of family CKD-05 had mild proteinuria (0.179 g/24 h) and significantly increased serum creatinine. She was presented with focal glomerulosclerosis (highly suspected FSGS) by renal biopsy. The two probands were both biopsy-proven FSGS (or highly suspected FSGS), but with different clinical features. One of the probands had more proteinuria and slightly decreased renal function, while the other one had less proteinuria but worse renal function. Although the mutation from the same amino site was reported before, there are several features indicating that our identified variant (c.T1655C:p.V552A) exhibited distinct clinical phenotypes that is likely also pathogenetic in our two affected families. Firstly, the identified mutation was not detected in 195 healthy people when Sanger sequencing was performed. Secondly, a good correlation between genotype and phenotype by segregation analysis further strengthens our findings. Thirdly, mutant XPO5 can cause podocyte dysfunction because our patients showed higher levels of proteinuria and glomerulosclerosis. Furthermore, the onset of diseases is different. The average onset age of our patients was 29.5 years while reported cases exhibited proteinuria at a very early age (2 years old). Lastly, our patients exhibited different inheritance pattern with autosomal dominant inheritance pattern, while in the previous study, the inheritance pattern was not determined. In addition, all clinical features suggested that heterozygosity of this variant would still be pathogenic, but just confer the later development of disease onset due to being lower gene expression level. Taking all the data together, we believe that the newly identified variant in these Chinese FSGS patients might represent distinct features with different pathogenesis mechanisms despite mutation from the same amino acid site. We will further explore the potential different mechanisms in future study.

The function of XPO5 has been associated with the SMAD signaling pathway in relation to kidney disease previously. Braun DA et al. [14] showed XPO5 colocalized in podocytes with synaptopodin, a marker of primary and secondary podocyte foot processes in adult rat glomeruli, in a pattern that has been described for many other products of genes if mutated, could cause SRNS. They described that other proteins like NUP93, importin7 and exportin5 (XPO5) could interact with SMAD proteins. Additionally, they demonstrated that mutations in NUP93 from individuals with SRNS abrogate the interaction with SMAD4 and importin7 by co-IP in HEK293 cells. Similarly, we suggest that our identified variant may affect SMAD pathway. As XPO5 was detected in developing podocytes in adult rat. Hence, any abnormality in XPO5 can cause podocyte injury, which is the major cause of proteinuria and glomerulosclerosis as described by Michio N [21]. Taking all together, genotype–phenotype correlation, conservation analysis, effect on the structure coordination among amino acids and prediction scores of the online tools affirm that our identified mutation is pathogenic.

Other variations at the same amino acid have been reported. Previously, a homozygous XPO5 (p.V552I) variant has been reported in a sporadic case with SRNS and speech development delay without any known inheritance pattern in a 2-year-old Turkish boy. Interestingly, another study [22] reported the same variant (p.V552I), as somatic mutation in the search of somatic mutation burden in a single case of chondrosarcoma along with other 38 mutations, but they did not mention zygosity. The onset age of the reported SRNS patient was early (2-year-old). The average onset age of our patients was 29.5 years, which is the supporting evidence of variable expressivity related to zygosity status. Hence, homozygous patient (Turkish boy) showed early onset age of the kidney dysfunction while heterozygous patients (our study) showed adult-onset age of the disease. Altogether, the onset age of the disease directly correlates with the status of the zygosity and expressivity. Additionally, a third variant on the same position (p.V552F) is present in some databases without any phenotype association.

In this study, the *in silico* analysis has also affirmed that the identified variant is pathogenic. In gnomAD database (v3.1.2), the variant is tolerated by SIFT prediction (score: 0.17), which is similar for p.V552I (predicted to be tolerated with a score of 0.16) but other prediction tools like CADD (24.3 vs. 22.9), Fathmm-XF (0.85 vs. 0.0057) and REVEL (0.217 vs. 0.194) showed higher scores for p.V552A than p.V552I (https://gnomad.broadinstitute.org/variant/6-43551371-A-G?dataset=gnomad_r3). They did not functionally characterize p.V552I variant, but they showed that C-terminally GFP-tagged NUP93 precipitates endogenous SMAD4. C-terminally GFP-tagged SMAD4 precipitates endogenous NUP93. Upon co-overexpression, GFP-tagged NUP93 interacts with Myc-tagged SMAD4. Mutations (Lys442Asnfs*14, Gly591Val, Tyr629Cys) identified in individuals with SRNS abrogate SMAD4 interaction. Upon BMP7 stimulation C-terminally GFP-tagged NUP93 interacts with phosphorylated/activated SMAD1/5. C-terminally GFP-tagged NUP93 precipitates endogenous importin7. C-terminally GFP-tagged SMAD4 precipitates endogenous importin7. Upon co-overexpression, GFP-tagged NUP93 interacts with Myc-tagged importin7. Mutations (Lys442Asnfs*14, Gly591Val, Tyr629Cys) identified in individuals with SRNS abrogate importin7 interaction. GFP-tagged SMAD4 precipitates endogenous XPO5. Deletion of the SMAD4 nuclear export signal (NES) (aa142-aa149) abrogates the interaction with XPO5. Altogether, all the proteins have very strong interaction and mutations in XPO5 can cause similar phenotypes as the patients having mutations in NUP93 (K442Nfs*14, G591V, Y629C). So we believe that our identified mutation can be pathogenic based on the higher score of prediction tools. To the best of our knowledge, our study describes the autosomal dominant inheritance pattern in adult-onset of familial FSGS/FGGS and the histopathological changes in association with *XPO5* variant for the first time.

The NPCs consist of highly conserved eukaryotic proteins known as NUPs within the nuclear envelope which mediate the nucleocytoplasmic transport of RNAs, ribonucleoprotein (RNP) and other small proteins by exportins and importins [6]. XPO5 plays a role in nucleocytoplasmic shuttling with the interaction of NUPs. Braun et al. have demonstrated that *XPO5* was present in developing podocytes along with NUP93 and NUP205 in fetal rat kidney at the capillary loop stage of renal glomerular development [14]. They also described that the defects in NUP93, NUP205 and XPO5 could affect SMAD signaling pathway, proving that SMAD signaling is the relevant pathogenic pathway for early onset SRNS in their patients with homozygous variants [14]. They also demonstrated the colocalization of XPO5 along with synaptopodin in adult rat glomeruli. Synaptopodin is a marker of primary and secondary podocyte foot processes and has been defined as a marker for some other products of genes in association with SRNS, if mutated [23–25]. Interestingly, all the patients having variants in the genes colocalizing with synaptopodin had homozygous variants and an early onset age of the disease [23–25].

FSGS can present with massive proteinuria with or without impaired renal function [20]. It is a leading cause of end-stage kidney disease. It accounts for about 20% of nephrotic syndrome in children. The renal biopsy of family FS-133’s proband showed FSGS and global sclerosis which were in accordance with his clinical features such as nephrotic range proteinuria and renal insufficiency. The renal biopsy of proband from CKD-05 family showed focal glomerulosclerosis (highly expected FSGS) with increased serum creatinine level.

Till date, few variants in exportins (only in *EXPO1* and *EXPO5*) have been reported and described in association with chronic lymphocytic leukemia, Wilms’ tumor, autism, multiple myeloma and minimal change nephrotic syndrome (MCNS) with SRNS, respectively [7, 9, 10, 26]. Moreover, Neggers JE et al. have demonstrated that a heterozygous variant confers a similar defect in Expo1 cargo as a homozygous substitution in human cell lines [27]. To the best of our knowledge, four variants have been reported in *XPO5,* of which only one homozygous variant has been identified in a boy with kidney disease and the other three variants have been found in patients with Wilms tumor, autism, and multiple myeloma. A homozygous variant in *XPO5* (c.G1654A, p.V552I) in a 2-year-old Turkish boy has been reported in association with SRNS and speech development delay, while renal biopsy showed MCNS [28]. Here, we implicate NPC-associated protein, *XPO5*, in the pathogenesis of FSGS in two Chinese families with different findings. The Turkish boy had a homozygous variant, while our patients have heterozygous. Furthermore, the Turkish boy exhibited MCNS at an early age (2 years), while our patients were adults and affected with focal glomerulosclerosis by renal biopsy. The Turkish boy was also primary SRNS and showed a response to CsA while our patient (the proband of family FS-133) showed only partial remission of proteinuria after immunosuppressant therapy including CTX [14]. Thus, our findings confirm that the identified variant was causing adult-onset biopsy-proven FSGS for the first time.

In this study, we performed direct sequencing of 40 adult-onset Chinese FSGS families, 77 sporadic FSGS cases and 157 CKD families (non-FSGS) and identified a novel heterozygous variant (c.T1655C:p.V552A) in *XPO5*. All the patients were carrying variants, while the normal individuals were wild type. Our findings showed that the identified variant in *XPO5* is causing adult-onset FSGS and its rate is low in Chinese familial FSGS (2.5%, 1/40) and in CKD (0.64%, 1/157).

Interestingly, the variant rate of NUPs was relatively lower in the Chinese population based on previous studies [28–32]. In contrast to prior studies related to NUPs variants, the *XPO5* variants discovered in families recruited in this study had heterozygous variant. The *in silico* analysis of the identified heterozygous variant in this study affirms that it is pathogenic and might cause the adult-onset FSGS/FGGS in our patients. Furthermore, we suggest the functional analysis of identified variant *in vivo* by using different model organisms. This study has two limitations, firstly the unavailability of validation at protein levels and secondly, the absence of the *in vivo* and *in vitro* functional evidence for the pathogenesis of the identified variant.

In summary, this is the first study that demonstrates a novel heterozygous variant of *XPO5* causing adult-onset of FSGS in two Chinese families segregating in an autosomal dominant pattern. Thereby, we describe a novel XPO5 protein to a new pathogenicity for FSGS. Additionally, the identification of the new inheritance pattern and the difference in onset age for *XPO5* can help us understand the recurrence risk within the families and the genetic counseling of such complicated impending nephropathies. This study will be helpful for nephrologists using genetic analysis to make the early diagnosis, disease management, future treatment and genetic counseling. Lastly, we implicate that SMAD signaling pathway has a high potential for new approaches toward FSGS therapy.

## Methods

### Subjects

We recruited a Chinese adult-onset FSGS cohort in this study, comprising a total of 40 FSGS families, 77 sporadic FSGS cases, 157 CKD families (non-FSGS) and 195 healthy controls. FSGS is defined by the presence of typical segmental sclerosis in some glomeruli in renal biopsy specimens. Familial FSGS is defined as one of the following conditions: (1) Two or more blood relatives diagnosed with FSGS by renal biopsy. (2) In addition to the proband who was diagnosed with FSGS by renal biopsy, at least one family member presented proteinuria (defined as urine protein equal or higher than +  + in urine routine test or urine protein equal or higher than 300 mg/d or urine albumin/creatinine ratio equal or higher than 10 mg/mmol), renal dysfunction (defined as EPI-eGFR less than 60 ml/min/1.73m^2^), or end-stage renal disease (ESRD) with unknown cause. Patients with secondary FSGS such as obesity, diabetic nephropathy, hypertensive nephropathy, or other heredity kidney diseases (such as Alport syndrome, thin basement membrane disease, and Fabry disease) were excluded. FSGS patients from this study have been recruited in our previous research, in which variants of *INF2* and *COL4A3* in Chinese FSGS patients have been reported [1, 19].

Clinical data such as creatinine, uric acid, urine protein and albumin were recorded from patients’ medical documents. Creatinine and uric acid were measured by enzymatic method. Albumin was measured by colorimetry. Urine proteins were measured by immunoturbidimetry. Estimated GFR was calculated using the CKD-EPI formula (2009) [33].

The proband of family FS-133 was presented with nephrotic range proteinuria and renal insufficiency with biopsy-proven FSGS. According to KDIGO practice guidelines on glomerulonephritis [34], he was treated with prednisolone (1 mg/kg/d) and an immunosuppressive agent (CTX and CsA). The proband of family CKD-05 had mild proteinuria and impaired renal function with focal glomerulosclerosis (highly suspected FSGS) in renal biopsy. According to KDIGO practice guidelines, renin–angiotensin–aldosterone system (RAS) blockade was started.

### Genotyping

WES was performed on probands of all FSGS families and all sporadic FSGS patients by BGI (Shenzhen, China) and CKD by Genergy Bio-Technology (Shanghai) Co., Ltd. First, the qualified DNA samples were randomly fragmented to generate 150–200 bp DNA fragments. The extracted DNA was amplified in a ligation-mediated polymerase chain reaction (LM-PCR). The Agilent SureSelect All Human Exome library was used to capture the exons of the human genome. High-throughput sequencing was performed on a Hiseq2000 platform (Illumina), and the sequence of each library was generated as 150 bp paired-end reads. We aligned the WES reads to the NCBI human reference genome (hg19) using Burrows–Wheeler Aligner (BWA). After marking the duplicated read pairs by Mark duplication from the Picard tools package, we called the germline SNV (Single Nucleotide Variants) and Indel (small insertions and deletions) using the Genome Analysis Toolkit (GATK). Annovar was used to annotate the variants. To obtain functional variants, we followed the filtration criteria given in Additional file 2: Fig S1. Afterward, the segregation of the identified variant and validation was done by Sanger sequencing within the families and 195 ethnically matched healthy controls, respectively. Polymerase chain reaction amplification was performed by using XPO5-1-F: 5'-CTGAGAAGATGGTAAGAAGGGC-3' and XPO5-1-R: 5’- GACTAGTAATTTGAGTCCAGCC-3' set of primers. The Sanger sequencing reactions were checked by agarose gel electrophoresis and sent to Shanghai Jie Li Biotechnology Co., Ltd. for sequencing results. BioEdit software editor, version 7.1 (www.mbio.ncsu.edu/BioEdit/bioedit.html) was used to compare wild-type and sequencing files of exon 15 of *XPO5* as obtained from the genetic analyzer.

### In silico structural prediction

The prediction of pathogenicity of the identified variant was done by online tools, such as MutationTaster (https://www.mutationtaster.org/), Polyphen-2 (http://genetics.bwh.harvard.edu/pph2/) and (http://fathmm.biocompute.org.uk/fathmm-xf/). The CADD-SV scores on the PHRED were measured by an online available tool (https://cadd.gs.washington.edu/snv). The effect of the variant on protein function and structure was also predicted by different tools. The XPO5 protein (UniProt ID Q9HAV4) structure was retrieved from the RSCB protein databank (https://www.rcsb.org/) which has PDB-ID 5yu6.1.A. The homology modeling was carried out by Swiss modeling (https://swissmodel.expasy.org). PyMol2 was used for visualization of protein structures and changes in hydrogen bonding (if any) due to variant. The structural changes were also predicted by the online analysis using HOPE (https://www3.cmbi.umcn.nl/hope/) and Missense3D (http://missense3d.bc.ic.ac.uk/~missense3d/).

## Supplementary Information

**Additional file 1: Table S1. **ACMG Criteria for identified variant.

**Additional file 2: Fig. S1. **Flowchart of whole-exome sequencing data analysis.

## Data Availability

All data included in this study are available upon request by contact with the corresponding author.

## Abbreviations

WES: Whole-exome sequencing
FSGS: Focal segmental glomerulosclerosis
CKD: Chronic kidney disease
*XPO5*: Exportin 5
FGGS: Focal global glomerulosclerosis
NPCs: Nuclear pore complexes
SRNS: Steroid-resistant nephrotic syndrome
AD: Autosomal dominant
AR: Autosomal recessive
ESRD: End-stage renal disease
GATK: Genome analysis toolkit
CTX: Cyclophosphamide
CsA: Cyclosporine A
ACR: Urinary albumin/creatinine ratio
MCNS: Minimal change nephrotic syndrome
