# Supplementary material for: Mutation in XPO5 causes adult-onset autosomal dominant familial focal segmental glomerulosclerosis
Source: Hum Genomics. 2022 Nov 12;16:57. doi: 10.1186/s40246-022-00430-y (PMC9655905; doi:10.1186/s40246-022-00430-y)
Supplement: Supplementary file 1 — Additional file 1: Table S1. ACMG Criteria for identified variant. [file 40246_2022_430_MOESM1_ESM.docx]

| **Table S1. ACMG Criteria for identified variant.** | | | | | | | | | | | | | | | | | | | |
| --- | --- | --- | --- | --- | --- | --- | --- | --- | --- | --- | --- | --- | --- | --- | --- | --- | --- | --- | --- |
| **Gene** | **c.DNA** | **Protein** | **PVS1** | **PS1** | **PS2** | **PS3** | **PS4** | **PM1** | **PM2** | **PM3** | **PM4** | **PM5** | **PM6** | **PP1** | **PP2** | **PP3** | **PP4** | **PP5** | **Variants class** |
| *XPO5* | c.T1655C | p.V552A | N | N | N | N | N | N | Y | N | N | Y | N | Y | N | Y | Y | N | LP |
| N, no; Y, yes; LP, likely pathogenic. | | | | | | | | | | | | | | | | | | | |
