# Supplementary figures and images for: Mutation in XPO5 causes adult-onset autosomal dominant familial focal segmental glomerulosclerosis
Source: Hum Genomics. 2022 Nov 12;16:57. doi: 10.1186/s40246-022-00430-y (PMC9655905; doi:10.1186/s40246-022-00430-y)

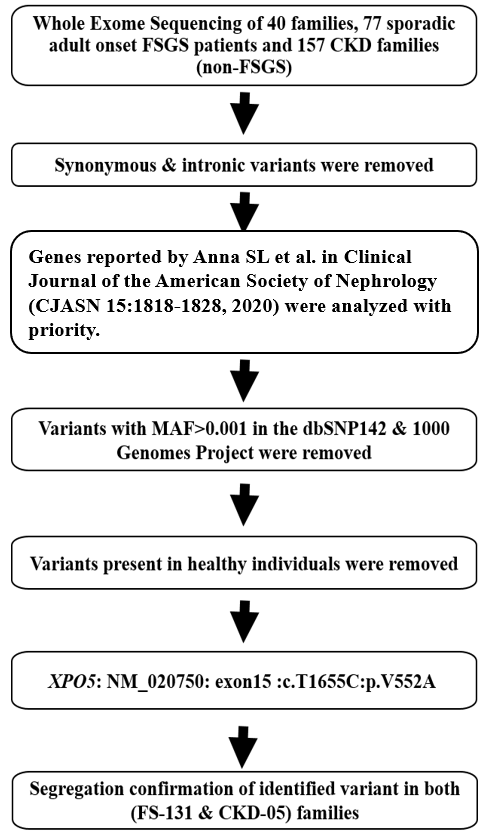
**Figure S1. Flow chart of whole-exome sequencing data analysis.**

Supplement: Supplementary file 2 — Additional file 2: Fig. S1. Flowchart of whole-exome sequencing data analysis. [file 40246_2022_430_MOESM2_ESM.docx]
